# Median raphe cyst with comet-tail artifacts on ultrasonography: A rare case report

**DOI:** 10.1016/j.jdcr.2025.07.029

**Published:** 2025-08-25

**Authors:** Taichi Imamura, Yukari Imamura, Chika Ohata

**Affiliations:** a Imamura Dermatology and Plastic Surgery Clinic, Ube, Japan; b Department of Dermatology, Osaka General Medical Center, Osaka, Japan

**Keywords:** comet-tail artifacts, median raphe cyst, ultrasonography

## Introduction

Median raphe cysts (MRCs) are rare benign cystic tumors occurring in males. This report presents an MRC displaying multiple internal hyperechoic foci with comet-tail artifacts on ultrasonography. To the best of our knowledge, only 1 similar case has been previously reported.^1^ This case highlights an uncommon ultrasonographic finding and considers possible mechanisms underlying its occurrence.

## Case report

**Fig 1:**
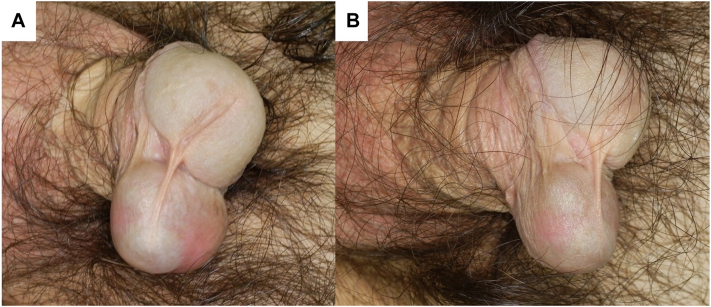
Clinical appearance of a subcutaneous tumor contiguous with the median raphe of the penis: **(A)** frontal view and **(B)** lateral view.

**Fig 2:**
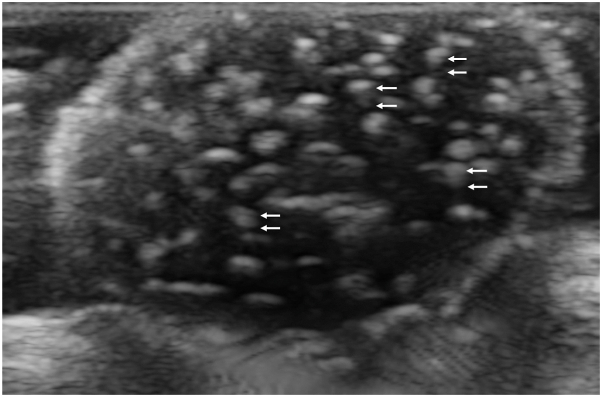
Ultrasonography image showing multiple bright spots with a comet-tail artifact. *White arrows* indicate the start and end points of the comet-tail artifact.

A 49-year-old male patient had been diagnosed with a subcutaneous penile tumor during childhood. The lesion gradually increased in size but remained untreated until pain associated with friction prompted presentation to the hospital. Clinical examination revealed a 3.0 × 2.5 × 2.4 cm^3^ subcutaneous mass located along the median raphe of the penis (Fig 1). The mass was freely movable, elastic, firm, and well circumscribed. Ultrasonography revealed a hypoechoic cystic lesion containing multiple internal echogenic foci with comet-tail artifacts (Fig 2). Surgical excision was performed under local anesthesia.

**Fig 3:**
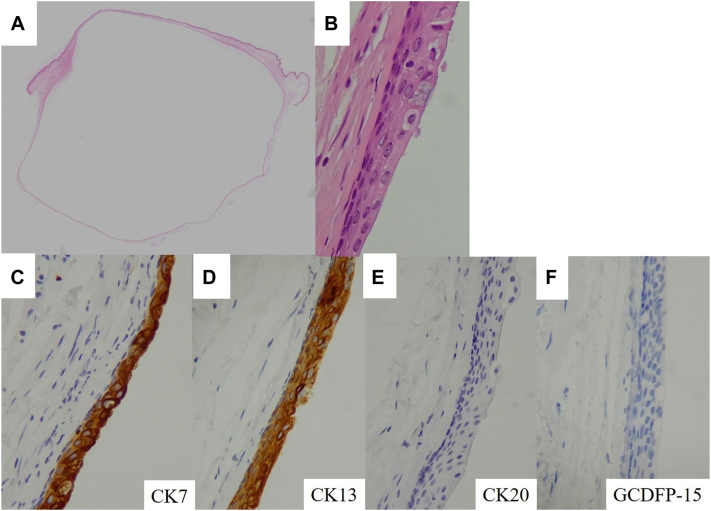
Histological and immunohistochemical analyses of the tumor. **A,** At low magnification, the tumor presents as a unilocular cyst lacking continuity with the overlying epidermis (hematoxylin and eosin stain [H&E], original magnification ×1). **B,** The cyst wall comprises stratified squamous epithelium and stratified columnar epithelium with glandular cells (H&E, ×400). **C**-**F,** Immunohistochemical staining showing that tumor cells are positive for CK7 and CK13, and negative for CK20 and GCDFP-15 (all ×200).

Gross examination revealed that the cyst was filled with a brownish gelatinous material. Histopathologic analysis demonstrated a unilocular cyst not continuous with the epidermis. The cyst wall was externally lined with stratified squamous epithelium and internally with stratified columnar epithelium containing glandular cells. Immunohistochemical staining showed positivity for CK7 and CK13, and negativity for CK20 and GCDFP-15, findings consistent with MRC (Fig 3). However, no definitive histopathologic features accounted for the internal hyperechoic foci observed on ultrasonography.

## Discussion

MRCs are uncommon cystic lesions located along the anogenital median raphe, extending from the perianal region to the glans penis. Their origins remain uncertain, and various etiologies have been proposed. The most widely accepted theory attributes their development to congenital anomalies, such as incomplete closure of the urethral groove or defective embryological closure of the median raphe.^2^, ^3^, ^4^ These cysts are typically small, ranging from 0.88 to 1.1 cm in size and rarely exceed 2 cm.^2^^,^^3^ On ultrasonography, they usually appear as anechoic or hypoechoic, well-circumscribed lesions without internal structures.^2^^,^^5^ Only 2 previous reports have documented internal hyperechoic spots,^1^^,^^6^ 1 of which demonstrated comet-tail artifacts.^1^ The underlying cause of these unusual ultrasonographic findings has not yet been determined.

One possible explanation may be inferred from comparisons with thyroid colloid cysts, which also demonstrate multiple hyperechoic foci with comet-tail artifacts on ultrasonography. These cysts contain thick colloid, a glycoprotein-rich material that can form minute crystals following inspissation—that is, the progressive condensation and thickening of gelatinous content.^7^ Reflection of ultrasound waves from these crystals produces bright echogenic foci.^8^ Subsequently, multiple reverberations between closely spaced reflective surfaces give rise to comet-tail artifacts on ultrasonography.^9^

In reported cases, including the present one, MRCs demonstrating hyperechoic spots were substantially larger (3.0-10.2 cm) than typical MRCs, indicating the possibility of excessive mucinous secretion.^1^^,^^6^ The brownish gelatinous material observed in the present case suggested highly concentrated secretion. It was, therefore, hypothesized that, similar to thyroid colloid cysts, excessive mucus production leads to the formation of microaggregates, resulting in echogenic foci with comet-tail artifacts. This phenomenon may not be exclusive to thyroid colloid cysts or MRCs but could potentially occur in any cystic lesion characterized by high-viscosity contents retained within a confined compartment. However, not all large MRCs exhibit this feature, indicating that other factors, such as differences in cyst composition, may contribute. In the present case, cyst contents were discarded before a detailed biochemical analysis could be conducted. Future studies should investigate the contents of MRCs exhibiting hyperechoic foci to elucidate the pathophysiological basis of this rare ultrasonographic feature.

## Conflicts of interest

None disclosed.
